# Supplementary material for: EvANI benchmarking workflow for evolutionary distance estimation
Source: Brief Bioinform. 2025 Jun 12;26(3):bbaf267. doi: 10.1093/bib/bbaf267 (PMC12159288; doi:10.1093/bib/bbaf267)
Supplement: Supplementary_Figures_bbaf267 [file supplementary_figures_bbaf267.pdf]

Supplementary Figures

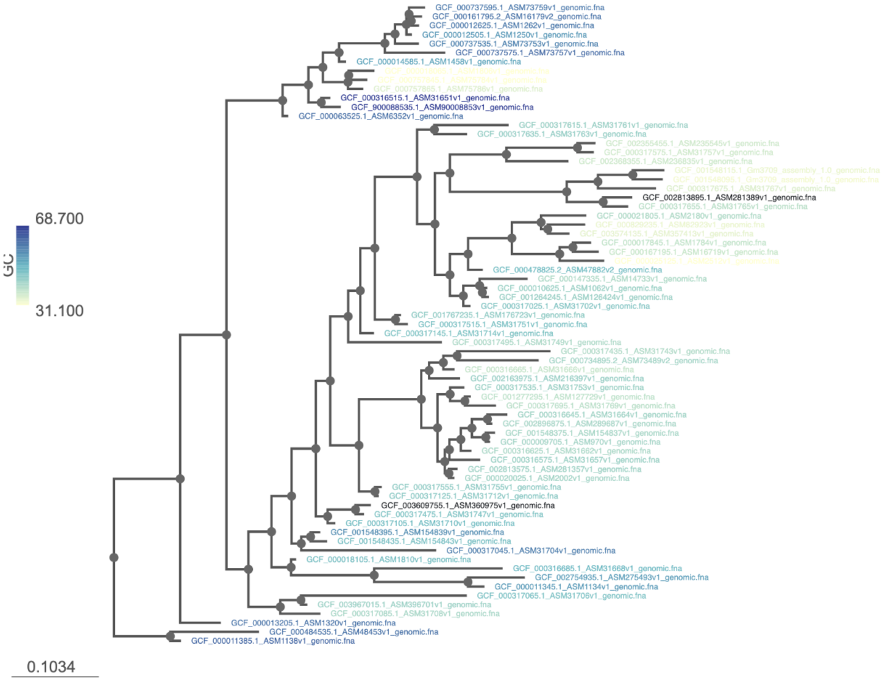

Supplementary Figure 1: The GC content of species in Cyanobacteriota. There is high variation in GC content in Cyanobacteriota, ranging from 31% to 68%.

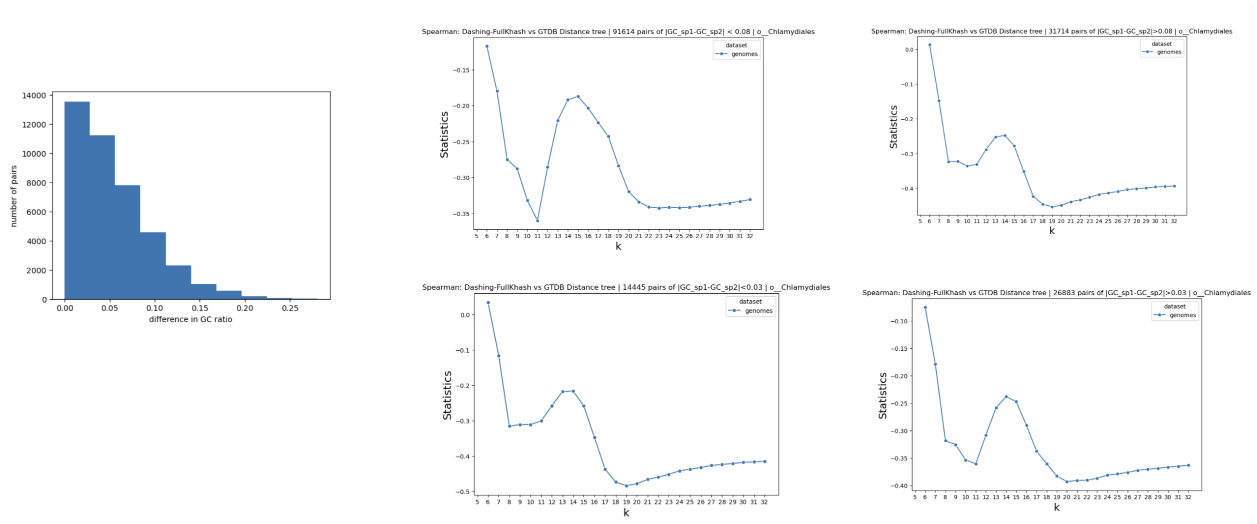

Supplementary Figure 2: (left) The histogram of difference in GC ratio between every species pair. (right) In each subplot, we keep only a portion of pairs. If the GC difference had been the cause (of two minima separate considering all pairs), the sub-figure for pairs with similar GC would not have had two minima. Thus, difference in GC content does not justify the two local minima seen in **Figure 3**.

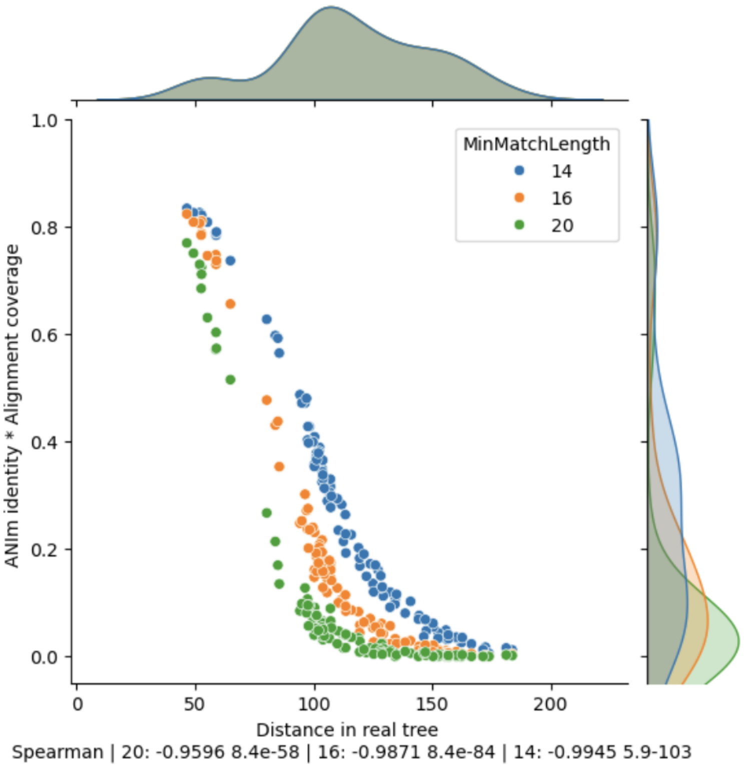

Supplementary Figure 3: Impact of changing the MUMmer parameter minimum match length in distance estimation.

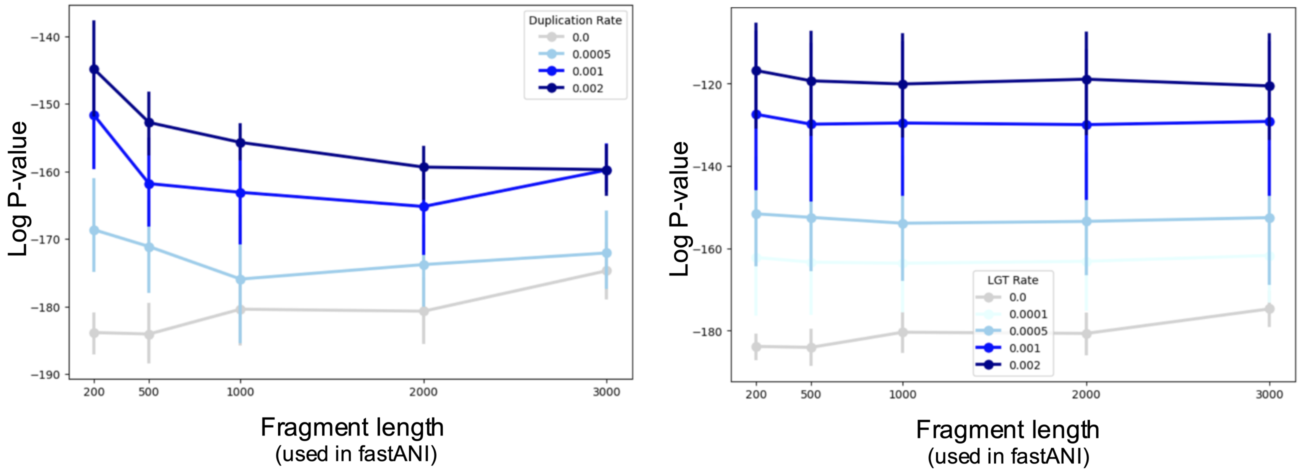

Supplementary Figure 4: Impact of changing fragment length used in FastANI across different simulated data varying duplication and LGT rates.

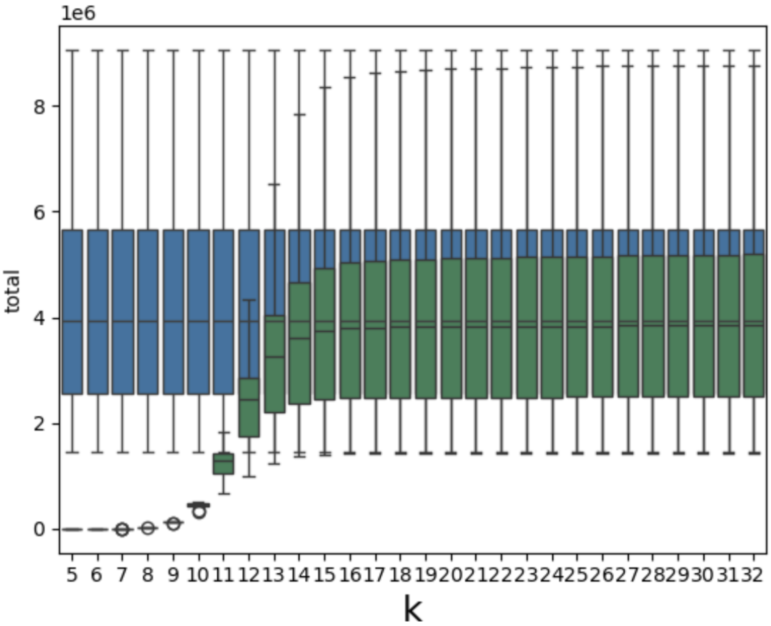

Supplementary Figure 5: (blue) total number of k-mers, which is (almost) equal to the genome size, (green): number of distinct k-mers found by the KMC tool. We can see there is a smooth increase in the number of distinct k-mers.

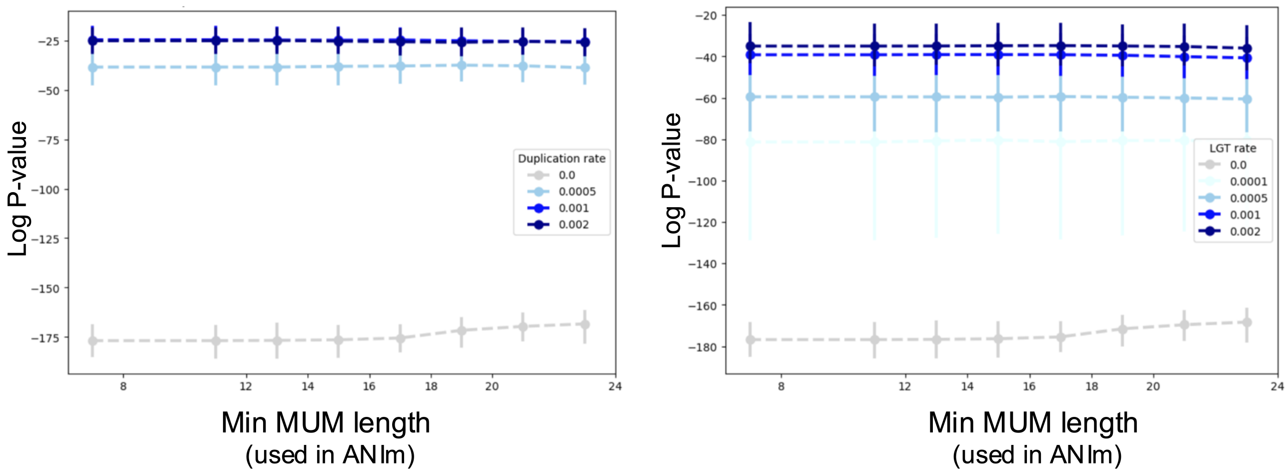

Supplementary Figure 6: The correlation between ANIm∗alignment fraction and tree distance for different minimum MUM length. The alignment fraction here refers to the amount after removing duplicates using `delta-filter -1` to keep only `1-1` alignments. In the last row we reported the alignment fraction before filtering. The simulated results show that the improvement for distant species achieved by weighted ANIm with the alignment fraction does not hold when there is high amount of duplications or LGT.

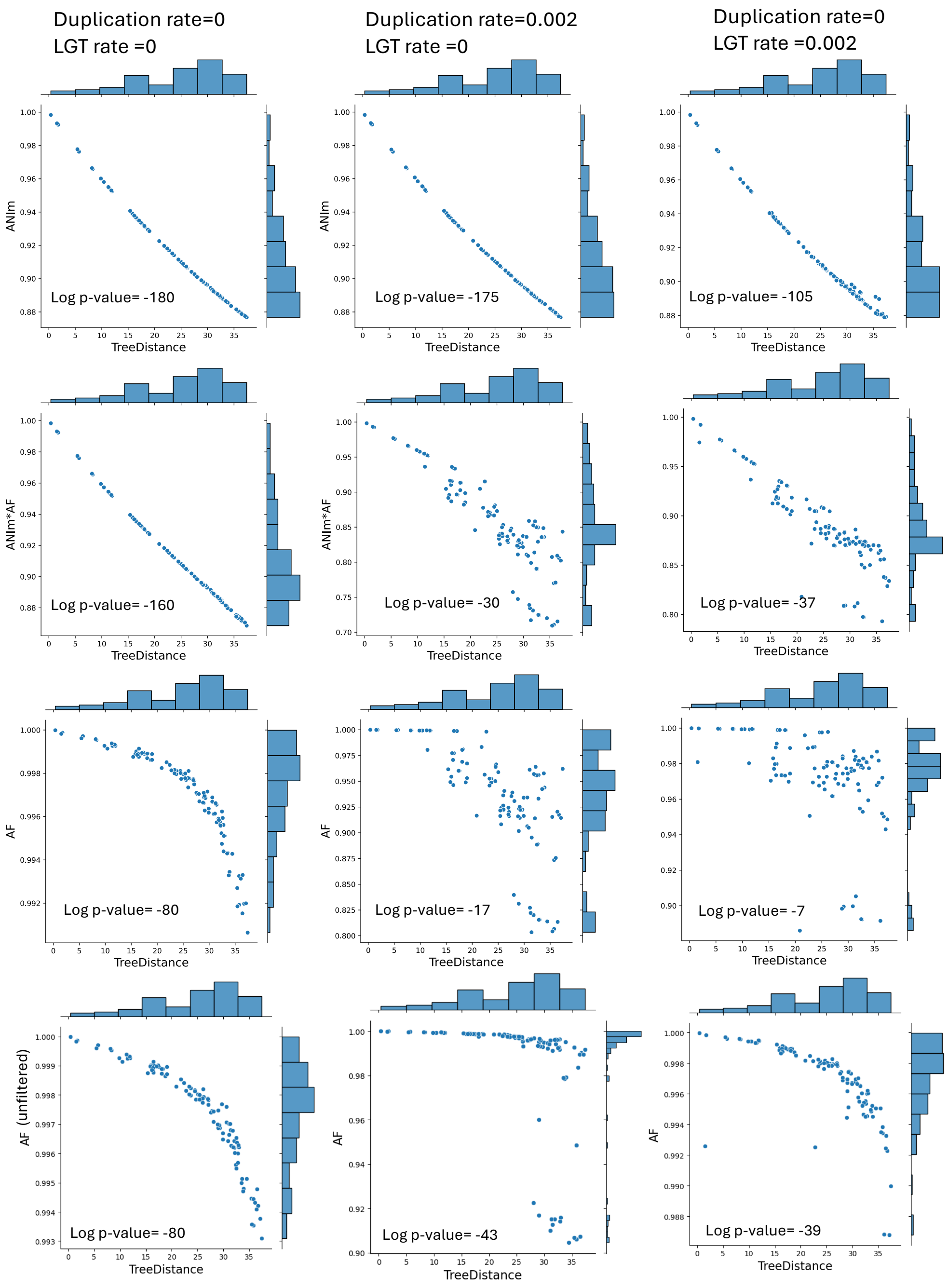

Supplementary Figure 7: The correlation between ANIm∗alignment fraction and tree distance is impacted by the fact that *AF* is not well correlated with distance when there is higher LGT or duplication. The reported log p-values in the figure are based on the Spearman correlation test. Each point is one of the 105 pairs for 15 simulated genomes. The last two rows correspond to alignment fraction after and before filtering using `delta-filter` to find 1-to-1 alignments. In all figures, NUCmer was executed in MUM mode.

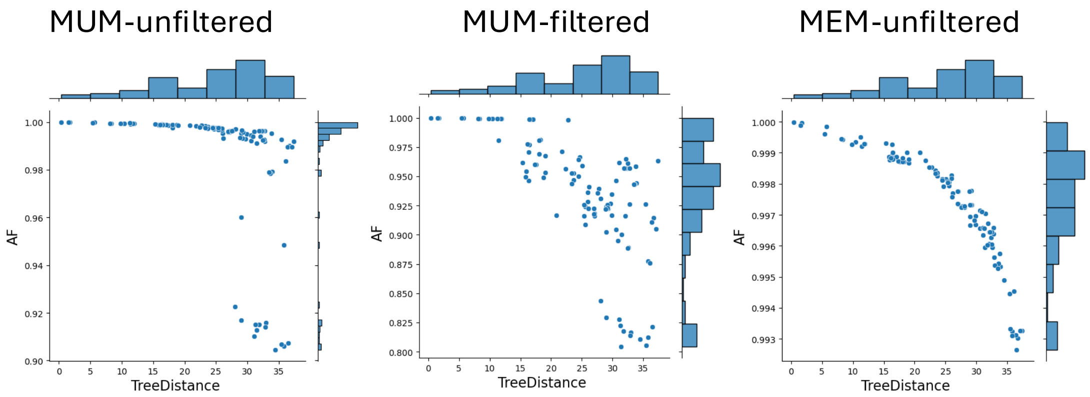

Supplementary Figure 8: The impact of using MEM (maxmatch) instead of MUM with NUCmer in ANIm. Keeping only 1-to-1 alignments (MUM-filtererd) using `delta-filter` also decreases the alignment fraction.

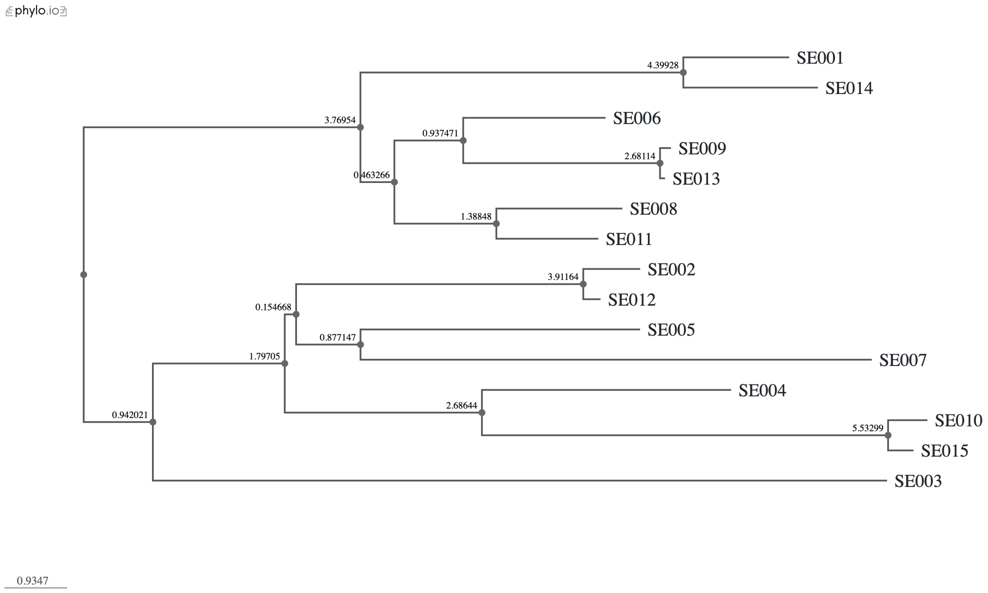

Supplementary Figure 9: An example tree output of the ALF simulator with branch length of 10. The tree includes 15 species (SE001..SE015).

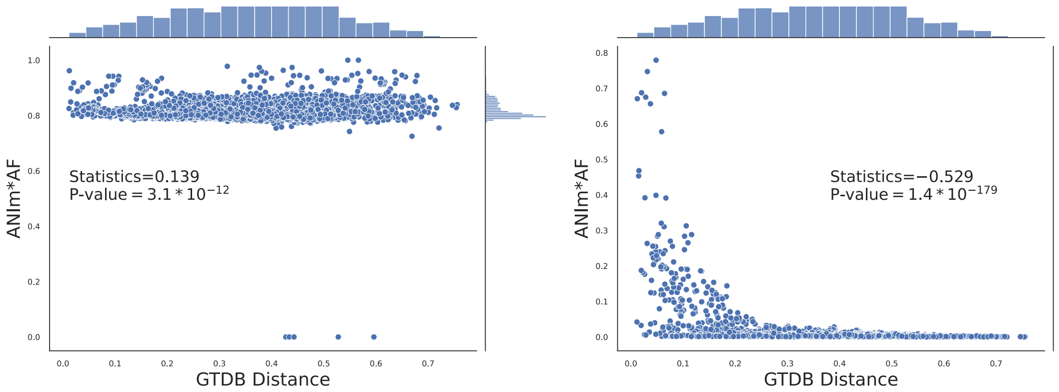

Supplementary Figure 10: Impact of weighting ANIm with alignment fraction (AF) for distance calculation in Cyanobacteraia, similar to Figure 8 but here GTDB tree is used instead of NCBI tree. Here, 71 species are considered and 13 species were discarded due to incompatibility of species names between GTDB and NCBI taxonomy. The same pattern is observed with more pronounced p-values.

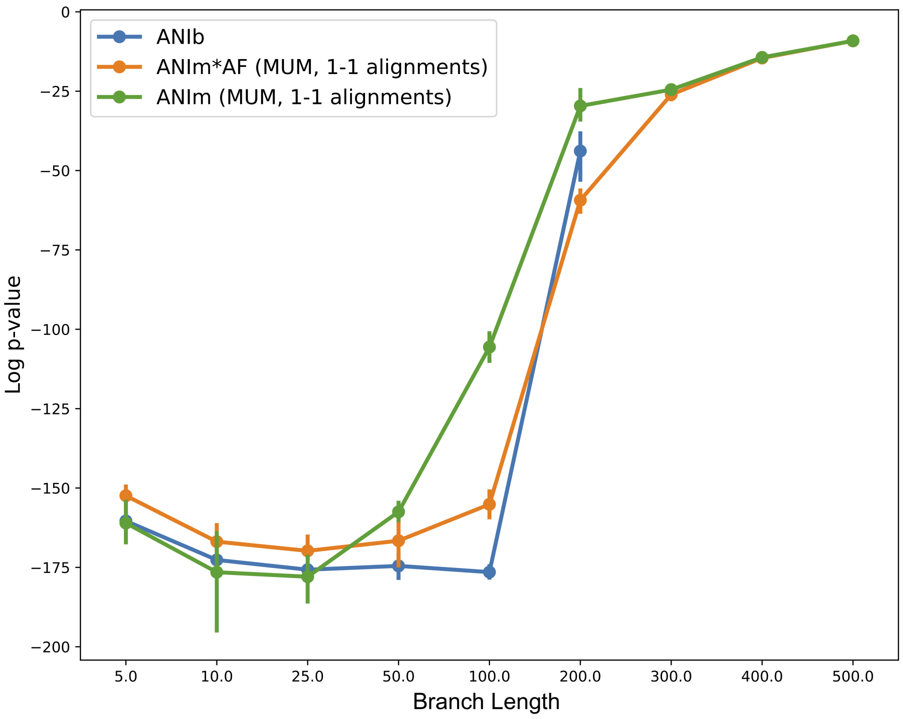

Supplementary Figure 11: Comparing ANIb with ANIm and $ANIm * AF$. Note that when branch length is very high (≥ 200), there is not much homology that can be detected by BLAST. BLAST output is empty for these cases when the e-value threshold was e^{-15}.

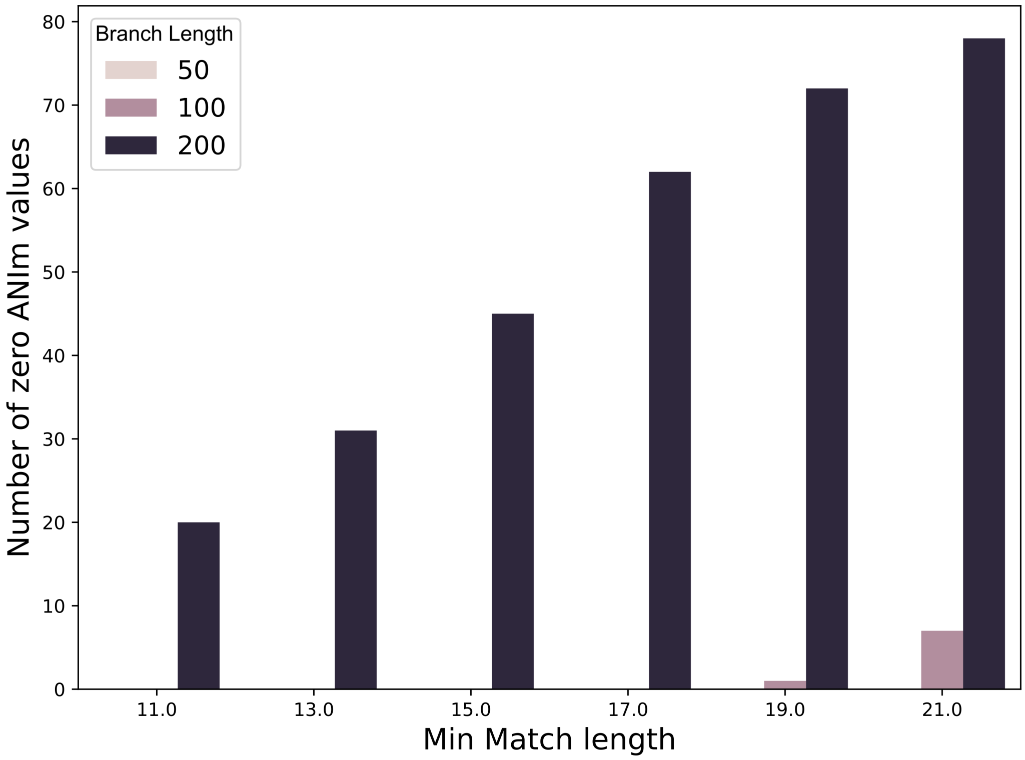

Supplementary Figure 12: Number of zero ANIm values increases by branch length (more divergent dataset), affecting the power of ANIm. This could be mitigated by decreasing the minimum match length, ultimately improving rank correlation between ANIm and distance tree (e.g. in **Supplementary Figure 3**).

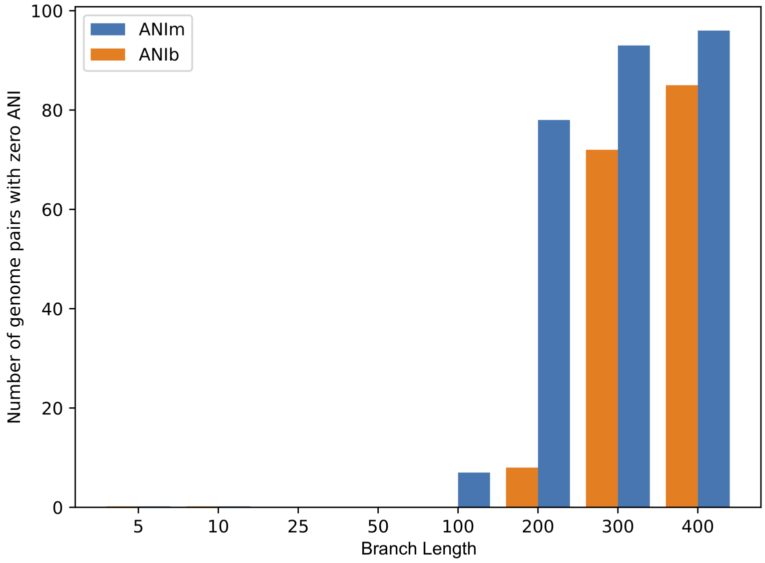

Supplementary Figure 13: Impact of genome divergence (Branch length) on number of zero ANIm and ANIb values. We can see ANIm tends to have more zeros compared to ANIb, specially for branch length greater thank 100.

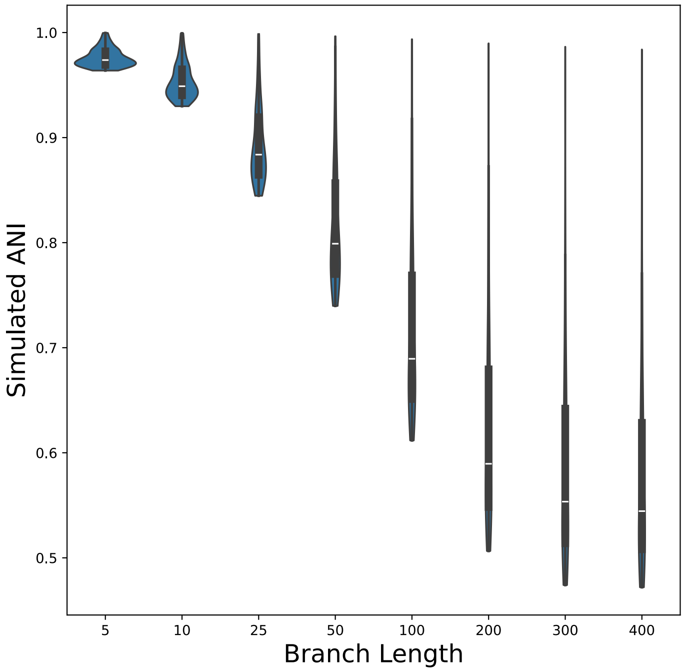

Supplementary Figure 14: Impact of branch length on the true (simulated) ANI found from the MSA generated by the ALF simulator.

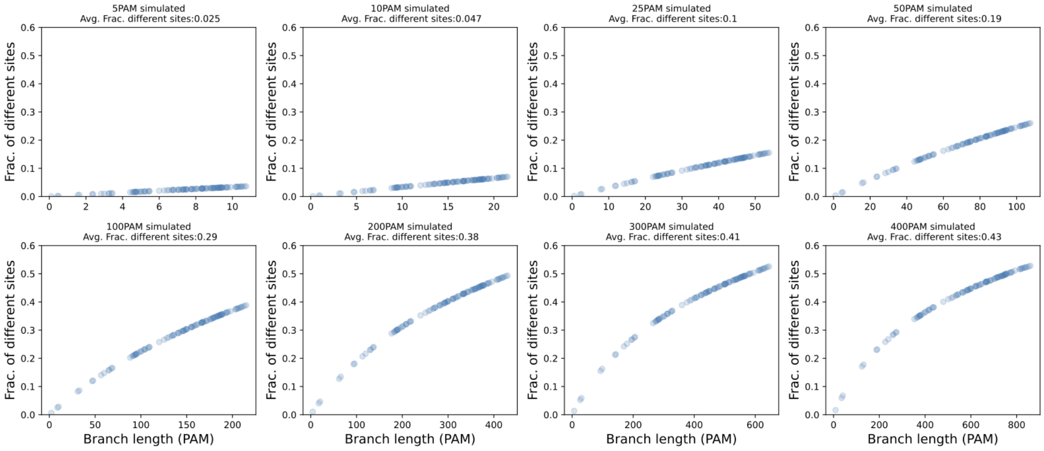

Supplementary Figure 15: Impact of changing the Branch Length (MutRate) parameter in ALF on simulated genomes reported as the fraction of different sites.

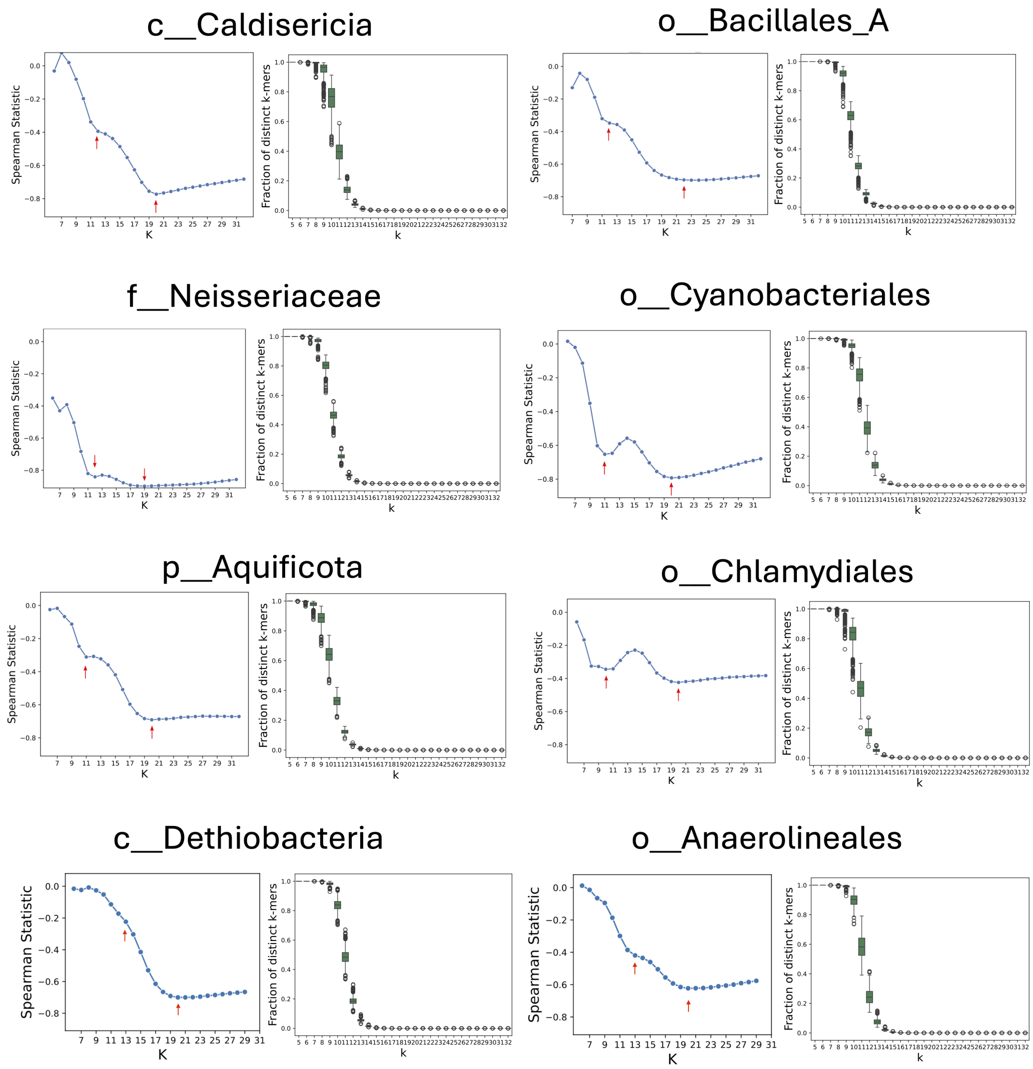

Supplementary Figure 16: A comparison of trends in Spearman rank correlation (Figure 3) and the fraction of distinct k-mers for eight clades. The fraction was calculated on 1000 genome pairs for each dataset.
